# Experience using 675 nm laser on three cases of Fitzpatrick skin type IV‐V with melasma

**DOI:** 10.1111/srt.13748

**Published:** 2024-05-20

**Authors:** Ruri D. Pamela, Massimo DF Vitale, Irene Fusco, Tiziano Zingoni, Kyu‐Ho Yi

**Affiliations:** ^1^ Department of Dermatology Venereology, & Aesthetic The General Soedirman National Defense Central Hospital Jakarta Indonesia; ^2^ Polyclinic of San Domenico Private Practice Bologna Italy; ^3^ Department of Clinical Research and Practice El.En. Group Calenzano Italy; ^4^ Division of Anatomy and Developmental Biology, Department of Oral Biology Yonsei University College of Dentistry Seoul South Korea

Dear Editor,

Melasma, predominantly affecting darker skin types, especially those within Fitzpatrick phototypes IV‐V.^1^ The treatment approach is further complicated by high recurrence rates and the significant impact melasma can have on patients' quality of life.^2^

Laser treatments are a significant component in the management of melasma, they are generally considered as second‐line treatments following the first‐line use of topical therapies. Many studies suggests that the low‐fluence Q‐switched Nd:YAG and picosecond laser are highlighted as potentially effective option in management of this condition especially when combined with other treatments.^3^ However, in darker skin types, there is a higher risk of post‐inflammatory hyperpigmentation (PIH) with laser and light treatments. Therefore, these treatments should be used with caution and potentially as a second‐line therapy after topical treatments.^3^, ^4^ A new non‐ablative laser with a wavelength of 675 nm has been found to significantly improve patients with melasma, which histologically in preclinical studies is shown by the presence of selective damage in melanin‐rich areas.^5^, ^6^ The aim of this article is to present the outcomes of a 675 nm laser treatment on patients with Fitzpatrick skin types IV‐V with melasma. By exploring the efficacy of 675 nm through these clinical cases, we seek to contribute to the limited pool of studies on laser treatments for skin of color and provide insights into the potential of this specific wavelength in managing melasma with minimal adverse effects.

This prospective case series involved three Indonesian female patients with Fitzpatrick Skin Types IV‐V, presenting with facial melasma. Exclusion criteria included hypersensitivity to light, use of photosensitizing agents, seizure disorders triggered by light, pregnancy, personal or family history of skin cancer, recent sun exposure, presence of tattoos or skin disorders in treated areas, and any melasma treatment within the previous 2 months.

The 675 nm laser system (RedTouch, Deka M.E.L.A., Calenzano, Italy) used in this study, was specifically chosen for its effectiveness in targeting melanin and stimulating collagen remodeling.^7^

The laser system was equipped with scanning shapes of square and it included the use of adjustable parameters: power, time exposure (dwell time), spot spacing, and an integrated skin cooling feature maintained at 5°C.

Before each treatment session, the patients' faces were cleansed with mild soap and water. The laser energy was adjusted according to the patient's skin type and tolerance, determined by a test area. The endpoint of the test was mild erythema and oedema, observable within 5–10 min. Each patient underwent three treatment sessions, spaced 1 month apart. The parameters for the laser treatment included power (5–7 W), dwell time (150 ms), spacing (2500 µm), stack 1 and integrated skin cooling (5°C). A transparent conductive gel was used to administer the treatment.

During treatment, the laser handpiece was passed over the skin surface in contact mode, ensuring consecutive spots without overlapping. Topical anesthesia was optional and, if used, was thoroughly removed before treatment. After treatment, the skin was cooled with cold water‐soaked gauzes. A nonsteroidal anti‐inflammatory cream containing vitamin B12 was applied twice daily for 2 weeks. Patients were instructed to use a total block mineral sunscreen throughout the treatment and follow‐up period.

The primary outcome was the change in melasma severity, assessed using the modified Melasma Area and Severity Index (mMASI) scores. Scores were recorded before the start of treatment and at 3 months after the last laser session. Digital photographs with skin analyzer (Janus, PIE, Gyeonggi‐do, South Korea) were taken before and after treatment to visually document changes. The safety profile was evaluated by monitoring for the appearance of side effects, including erythematous reactions, blistering, scarring, burns, hypopigmentation, or hyperpigmentation.

All three patients demonstrated significant clinical improvement in their melasma, as indicated by the reduction in modified Melasma Area and mMASI scores. The scores were reduced as follows:

Case 1: A 39‐year‐old female, initial mMASI score of 5.9, reduced to 3.4 after three sessions.
Case 2: A 47‐year‐old female, initial mMASI score of 7.3, reduced to 2.4 following treatment.
Case 3: A 49‐year‐old female, starting with a mMASI score of 4.0, which lowered to 2.8 after completing the sessions.

Digital photographs with skin analyzer taken before and after treatment (Figures 1, 2, and 3) showed noticeable improvement in the appearance of melasma. The photographic evidence is consistent with the quantitative mMASI score reductions, confirming the treatment's effectiveness.

**FIGURE 1:**
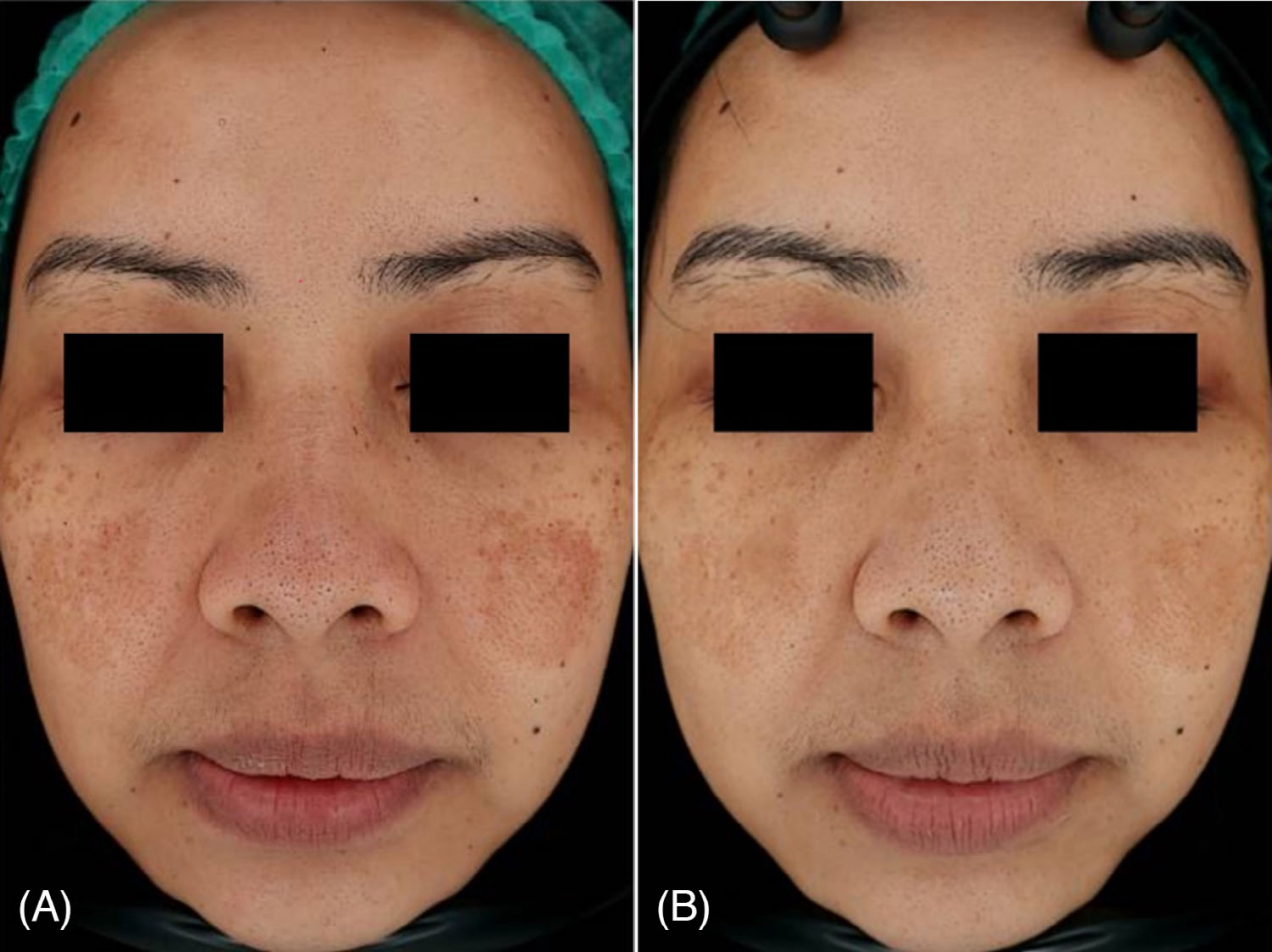
Clinical case of a 39‐year‐old female patient before (A) and at 3 months follow up after the last laser treatment session (B). A visible reduction of sign of facial melasma was observed.

**FIGURE 2:**
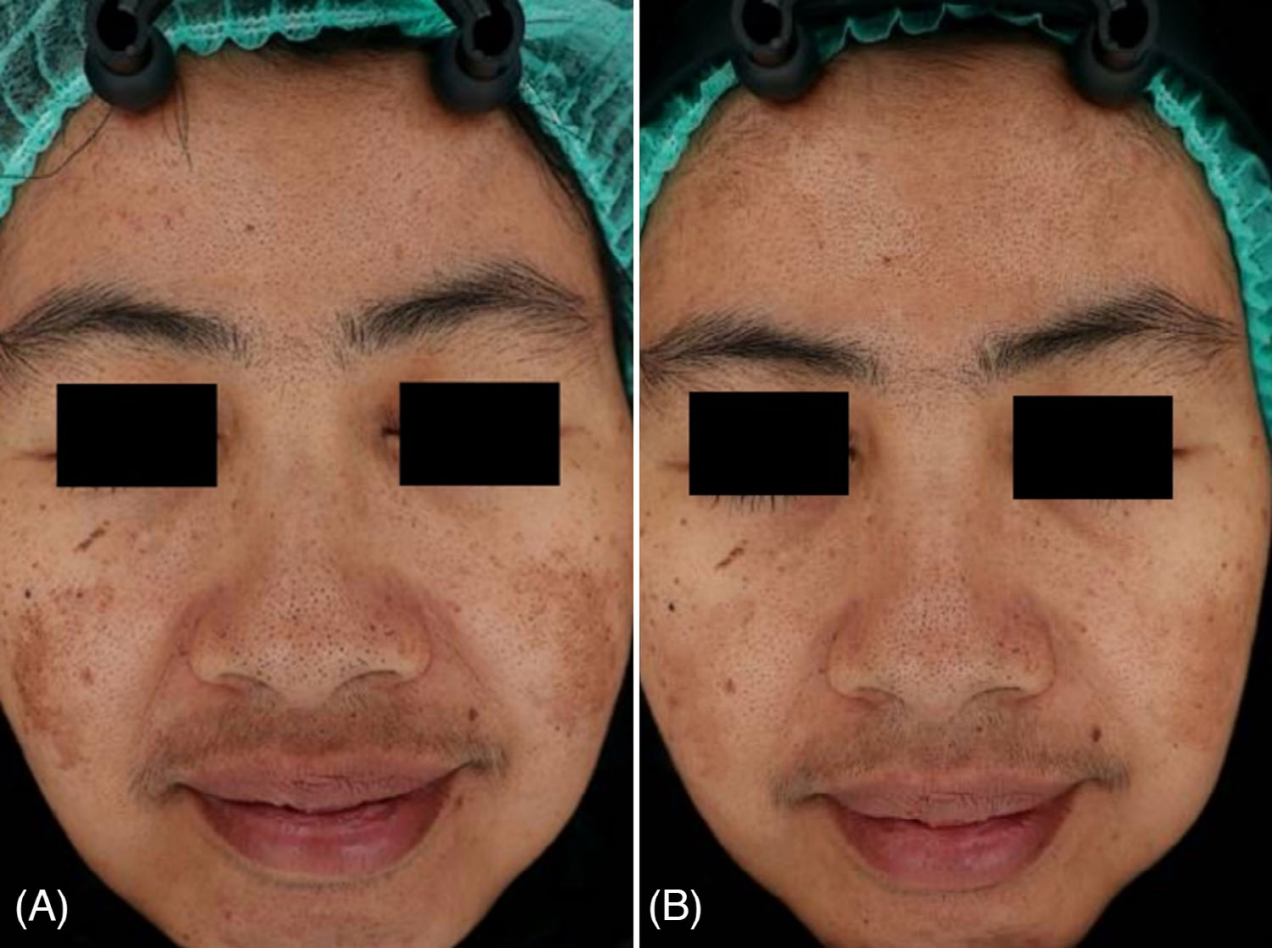
Clinical case of a 47year‐old female patient before (A) and at 3 months follow up after the last laser treatment session (B). A visible reduction of sign of facial melasma was observed.

**FIGURE 3:**
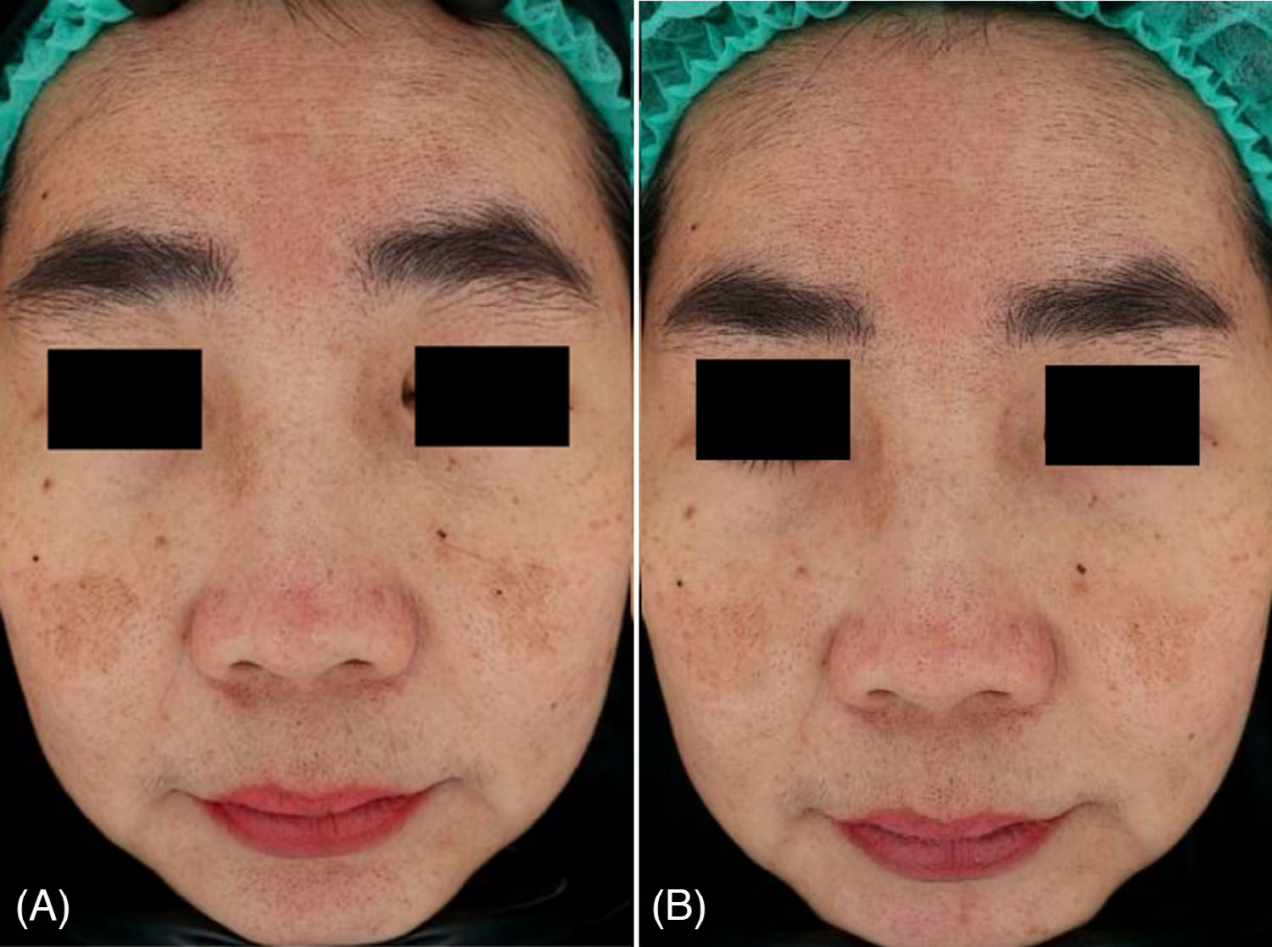
Clinical case of a 49year‐old female patient before (A) and at 3 months follow up after the last laser treatment session (B). A visible reduction of sign of facial melasma was observed.

Throughout the study duration, no serious adverse effects were reported, confirming the safety profile of the 675 nm laser for treating melasma in Fitzpatrick skin types IV‐V. Minor side effects included transient erythema, which resolved without intervention. No instances of blistering, scarring, or post‐inflammatory hyperpigmentation were observed. This safety profile was in line with expectations for this laser type and settings.

An informal assessment of patient satisfaction indicated high levels of satisfaction with the treatment outcomes. Patients reported improvements in the appearance of their skin and expressed satisfaction with the ease and comfort of the treatment process. The absence of significant downtime and the minimal side effects contributed to the overall positive treatment experience.

The 675 nm laser demonstrates significant potential in the treatment of melasma, especially in darker skin types. Studies have shown its efficacy in pigmentary improvements, marking it as an effective option for managing this condition​​.^8^ This aligns with the observed outcomes in the case series involving patients with Fitzpatrick skin type IV‐V, where notable improvements in melasma lesions were documented. The reduction in modified Melasma Area and mMASI scores across all cases underscores the laser's effectiveness in addressing hyperpigmentation​​. Additionally, the safety profile of this laser in darker skin types is corroborated by research highlighting its low interaction with hemoglobin, thereby minimizing risks associated with laser treatments in such skin types​​​​.^6^, ^9^

Red‐spectrum emission in this wavelength effectively targets melanin and collagen fibers, showing a strong affinity for these chromophores. In contrast, it interacts minimally with the vascular chromophore.^10^ Histologically, the 675 nm laser is observed to induce remodeling in collagen, promoting the proliferation of new collagen fibers. This plays a crucial role in treating melasma, as the interaction of the laser with collagen and melanin is pivotal in managing hyperpigmentation​​.^5^, ^9^ The treatment's effectiveness is not limited to superficial improvements but extends to substantial dermal changes, as evidenced by increased collagen production and rearrangement in treated areas compared to untreated ones. Such histological evidence provides a deeper understanding of the laser's mechanism of action, contributing to its efficacy in treating melasma​​.^9^

While the effectiveness and safety of the 675 nm laser for treating melasma in darker skin types are promising, considerations regarding treatment protocols are crucial. Adjustments in energy levels and careful monitoring are essential to cater to individual patient needs and minimize the risk of post‐inflammatory hyperpigmentation, especially in darker skin types​​.^6^, ^7^ Furthermore, the patient's feedback and satisfaction with the treatment, as well as the absence of significant downtime, contribute positively to the overall treatment experience​​.

The use of a 675 nm laser presents a promising and effective treatment for melasma in Fitzpatrick skin types IV‐V. The case series referenced indicate significant improvements in hyperpigmentation, as evidenced by the reduction in mMASI scores and enhanced skin appearance. Importantly, its safety profile is well‐suited for darker skin types, reducing the risk of adverse effects such as post‐inflammatory hyperpigmentation. While the initial results are encouraging, ongoing research and larger‐scale studies are essential to further validate these findings and optimize treatment protocols for broader application and long‐term effectiveness in managing melasma in individuals with darker skin type.

## AUTHOR CONTRIBUTIONS

Conceptualization, Ruri D. Pamela, Massimo DF. Vitale, Kyu‐Ho Yi; methodology, Ruri D. Pamela, Massimo DF. Vitale, Kyu‐Ho Yi; software, Ruri D. Pamela, Massimo DF. Vitale, Kyu‐Ho Yi; validation, Ruri D. Pamela, Massimo DF. Vitale, Kyu‐Ho Yi, Tiziano Zingoni; formal analysis, Ruri D. Pamela, Massimo DF. Vitale, Kyu‐Ho Yi; investigation, Ruri D. Pamela, Massimo DF. Vitale, Kyu‐Ho Yi; re‐sources, Ruri D. Pamela, Massimo DF. Vitale, Kyu‐Ho Yi; data curation, Ruri D. Pamela, Massimo DF. Vitale, Kyu‐Ho Yi; writing—original draft preparation Ruri D. Pamela, Massimo DF. Vitale, Kyu‐Ho Yi; writing—review and editing, Ruri D. Pamela, Massimo DF. Vitale, Kyu‐Ho Yi, Tiziano Zingoni, Irene Fusco; visualization, Ruri D. Pamela, Massimo DF. Vitale, Kyu‐Ho Yi, Tiziano Zingoni, Irene Fusco; supervision, Ruri D. Pamela, Massimo DF. Vitale, Kyu‐Ho Yi, Tiziano Zingoni, Irene Fusco; project administration, Ruri D. Pamela, Massimo DF. Vitale, Kyu‐Ho Yi, Tiziano Zingoni; funding acquisition, Ruri D. Pamela, Kyu‐Ho Yi, Tiziano Zingoni; All authors have read and agreed to the published version of the manuscript.

## CONFLICT OF INTEREST STATEMENT

Authors T.Z. and I.F. were employed by El.En. Group. The remaining authors confirm that no commercial or financial relationships that could be construed as a potential conflict of interest existed during the research.

## ETHICAL APPROVAL

The article is in accordance with the Declaration of Helsinki on Ethical Principles for Medical Research involving human subjects. Ethical approval is not necessary as the study device is already CE marked since 2019.

## Data Availability

The data that support the findings of this study are available from the corresponding author upon reasonable request.
